# Photo Quiz

**DOI:** 10.3201/eid1712.110484

**Published:** 2011-12

**Authors:** Myron G. Schultz, Peter Schantz

**Affiliations:** Author affiliation: Centers for Disease Control and Prevention, Atlanta, Georgia, USA (M.G. Schultz)

**Keywords:** veterinary epidemiology, zoonoses, One Medicine, Calvin W. Schwabe

**Figure:**
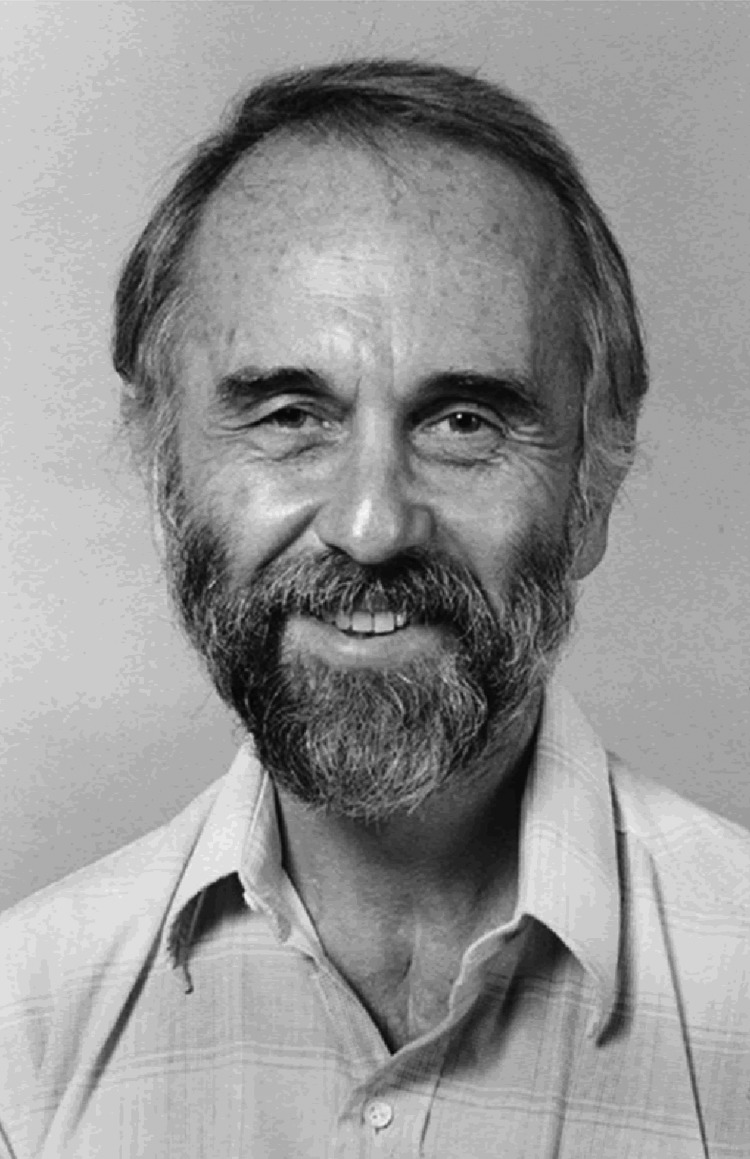
Who is this man?

Here is a clue: He founded the discipline of veterinary epidemiology and the concept of One Medicine.Is he:

- Martin Kaplan
- John McFadyean
- Karl F. Meyer
- Calvin W. Schwabe

Decide first, then turn the page.

## References

[1] Schwabe CW. Veterinary medicine and human health, 3rd ed. Philadelphia: Williams and Wilkins, Philadelphia; 1984.

[2] Schwabe CW. Unmentionable cuisine. Charlottesville (VA): University of Virginia Press; 1979.

[3] Schwabe CW. Cattle, priests, and progress in medicine (The Wesley W. Spink lectures on comparative medicine). Minneapolis: University of Minnesota Press; 1978.

[4] Schwabe CW. Epidemiology in veterinary practice. Philadelphia: Lea and Febiger; 1977.

[5] Schwabe CW. What should a veterinarian do (the challenge to young veterinarians, veterinary students, and persons who want to become veterinarians). Davis (CA): Centaur Press; 1972.

