# Supplementary material for: Photo Quiz
Source: Emerg Infect Dis. 2011 Dec;17(12):2365–7. doi: 10.3201/eid1712.110484 (PMC3311210; doi:10.3201/eid1712.110484)
Supplement: Related texts

# Other: Calvin W. Schwabe

**DOI:** 10.3201/eid1712.110484

**Authors:** Myron G. Schultz, Peter Schantz

**Affiliations:** Author affiliation: Centers for Disease Control and Prevention, Atlanta, Georgia, USA (M.G. Schultz)

This is a photograph of Calvin W. Schwabe (1927–2006). In a career that spanned 52 years, Schwabe made valuable contributions to a broad array of subjects, including epidemiology, zoonotic diseases, interactions of veterinary and human medicine, public health practice, livestock health in pastoral societies, ancient origins of human and veterinary medicine, and the philosophy of science. Calvin Schwabe is best known as one of the two 20th century founders of the discipline of veterinary epidemiology and the concept of One Medicine (now commonly referred to as One Health). The other founder of veterinary epidemiology and the One Medicine concept is James H. Steele, who worked at the Communicable Diseases Center (now Centers for Disease Control and Prevention) in Atlanta, Georgia.

Calvin W. Schwabe was born on March 15, 1927, in Newark, New Jersey. He graduated in 1948 with a BS in biology from Virginia Polytechnic Institute. In 1950, he received an MS in Zoology from the University of Hawaii. In 1954, Schwabe was awarded a DVM (with highest honors) from Auburn University, and in 1955, he received an MPH in tropical public health from Harvard University. His ScD in parasitology–tropical public health was awarded by Harvard in 1956.

From 1956 until 1966, Dr. Schwabe was a member of the medical and public health faculties of the American University of Beirut. There he developed a notable research program on hydatid disease and other parasitic zoonoses and founded a joint Department of Tropical Health within the medical and public health faculties and a Department of Epidemiology and Biostatistics within the School of Public Health. Beginning in 1960, he served as a consultant to the World Health Organization, developing a collaborative global program on hydatid disease research and control. During 1964–1966, he directed several parasitic disease programs at the World Health Organization headquarters in Geneva, Switzerland.

In 1966, Schwabe established the Department of Epidemiology and Preventive Medicine at the University of California, Davis, School of Veterinary Medicine, the first of its kind in the world at a veterinary school. He also established the Master of Preventive Veterinary Medicine degree program that teaches the principles and strategies of population-based medicine, disease surveillance, and prevention and control of diseases of food animals and zoonotic diseases that affect humans and animals. Today, his graduate students populate leadership positions in public health and research in many universities and government agencies.

In his masterwork, Veterinary Medicine and Human Health, first published in 1969, as well as in his many other writings, Schwabe promoted the concept of One Medicine, that is, stronger links and collaborations between the fields of human and veterinary medicine. Schwabe believed that the critical needs of humanity include combating diseases, ensuring sufficient and safe food, improving environmental quality, and creating a society in which humane values prevail. He saw the world as an ecosystem of interdependent civilizations and cultures in which human progress is inexorably linked with the co-evolution of the animal kingdom. Schwabe coined the term One Medicine, a concept previously expressed by Rudolph Virchow and William Osler, to focus attention on the commonality of human and veterinary health interests. One Medicine is the science of health and disease in which differences between humans and animals are not considered. Schwabe pointed out that most infectious diseases of humans have an animal origin. Schwabe also recognized the essential role of veterinary medicine in advancing medical research. Because human medical progress had been so dependent on veterinary studies, he urged schools of veterinary medicine to assume a much greater role in the training of scientists to conduct research to improve human health. He outlined specific reforms in the curricula of schools and colleges of veterinary medicine that would provide for the education of medical investigators.

Disciples of Schwabe and Steele have taken the One Medicine concept and advanced it into the One Health Initiative. The One Health Initiative is a movement that seeks to forge greater collaboration between the health disciplines, following the paths created by Schwabe and Steele. For example, at the University of California, Davis, School of Veterinary Medicine, where Schwabe taught for 25 years, the Calvin Schwabe One Health Project seeks to produce a new generation of veterinarians who will be ready to lead collaborative, multidisciplinary efforts to improve global health. With expertise in biohazard events, food and water safety, vector-borne diseases, established and emerging zoonotic diseases, herd health, foreign animal risks, and public health issues such as antimicrobial drug resistance, graduates of the program will be positioned as true One Health advocates and practitioners of the future.

In his book Cattle, Priests, and Progress in Medicine, Schwabe shows the link between animals and humans in medical progress by recounting highlights in the history of medicine since ancient times. He describes the early history of humans in terms of animal cultures, focusing on the ancient Nile Valley, and points to similarities in medical knowledge between present-day so-called cattle societies in northeastern Africa and the ancient people of the Nile. He discusses the comparative healers of ancient Egypt, the founders of Greek medicine, the Arabic contribution, and the beginnings of modern medicine. Schwabe shows that over the centuries many of the most notable breakthroughs in improving human health have been closely associated with observations and experiments on animals other than humans.

In Unmentionable Cuisine, Schwabe looks at food prejudices or why we eat what we eat, why we reject certain food sources as unpalatable, and the nutritional value of all food sources. He also introduces his readers to a vast number of authentic recipes, collected from all over the world, including recipes for preparing bugs, cow eyes, fertilized duck eggs, dog stew, and armadillo and turkey testicles, that are staples in some cultures but abhorrent to persons in other cultures.

Cal Schwabe was a man of great charm and intellect. He had many colleagues and admirers throughout the world. He belonged to the small class of people who are givers rather than takers—fountains rather than wells. Cal Schwabe was a Renaissance man, not merely because of his wide-ranging scholarly interests, but because he created a real renaissance in 20th century public health by showing the way to the unity of veterinary and human medical sciences.

## References

[1] Schwabe CW. Veterinary medicine and human health, 3rd ed. Philadelphia: Williams and Wilkins, Philadelphia; 1984.

[2] Schwabe CW. Unmentionable cuisine. Charlottesville (VA): University of Virginia Press; 1979.

[3] Schwabe CW. Cattle, priests, and progress in medicine (The Wesley W. Spink lectures on comparative medicine). Minneapolis: University of Minnesota Press; 1978.

[4] Schwabe CW. Epidemiology in veterinary practice. Philadelphia: Lea and Febiger; 1977.

[5] Schwabe CW. What should a veterinarian do (the challenge to young veterinarians, veterinary students, and persons who want to become veterinarians). Davis (CA): Centaur Press; 1972.
